# On Ascending Currents in Mucous Canals, and Gland Ducts, and Their Influence on Infection
*The Address in Surgery delivered at the seventy-third annual meeting of the British Medical Association by Charles John Bond, F.R.C.S., Eng.


**Published:** 1905-08-05

**Authors:** 


### ON ASCENDING CURRENTS IN MUCOUS CANALS, AND GLAND DUCTS, AND THEIR INFLUENCE ON INFECTION.*

Mr. Bond's address in surgery is a feature which alone will be sufficient to distinguish this year's meeting of the British Medical Association from its fellows of other years. In place of generalities and retrospective summaries, Mr. Bond chose for the theme of his address a subject which probably has never occurred to the majority of medical men in a definite form, although, upon reflection, it appears likely to prove a matter of the highest importance. He asks the question, "Do ascending currents occur in mucous canals and gland ducts"? By this Mr. Bond does not refer to the possibility of reflex peristalsis, but to the conveyance of minute particles along mucous canals and gland ducts in a direction contrary to that in which the chief contents of such ducts normally travel. As the result of several experiments, he is able to answer the question in the affirmative.

### The Large Intestine.

In cases where inguinal colotomy had been performed, or where a fistula was present leading to the large intestine, the insertion of a suppository containing indigo or carmine into the rectum was followed, after an interval of 12 hours or more, by the appearance of the pigment in the artificial opening. In one instance, that of a cæcal fistula following appendicitis, the introduction of indigo suppositories in the rectum on three successive days resulted in the appearance of indigo in the fistula, although the bowels were freely opened per anum in the intervening period. From these cases it appears: first, that in the large intestine, in cases in which the fæcal stream has been diverted by a previous colotomy, there is an upward current capable of carrying foreign particles, such as powdered indigo, along the bowel, and taking on the average about 24 hours to pass from the anus to the colotomy opening; secondly, that this current is not arrested by the presence of a stricture in the rectum, provided that there is some patency of channel left; thirdly, that it is not essential for this upward current that the fæcal current should be wholly diverted or absent; fourthly, that the ascending current takes about three days to pass from the anus along the whole length of the large intestine to the cæcum.

In attempting to explain this upward carriage of pigment Mr. Bond calls attention to the following points:—(1) That in the case of indigo the particles are insoluble in the intestinal juices; (2) that the current is not present in the bowel of a dead animal; (3) that, even in healthy animals, in the absence of any fistulous opening, there is some upward flow, though slight, along the bowel. He concludes that a consideration of the facts strongly suggests the explanation that the particles of indigo are carried up the bowel by a mucous current, and that while there is a central downward current of fæces, caused by peristaltic contraction, there is going on at the same time intermittently, or possibly alternating with the downward peristalsis, an upward peripheral mucous stream, covering and hugging the intestinal mucous membrane, and capable of conveying minute particles along with it.

### The Female Generative Tract.

In the case of the female generative tract more numerous opportunities occurred for careful observation. In a number of instances, previous to operations upon the uterine adnexa, a little sterilised indigo or carmine was placed upon, or just within, the os uteri. Careful microscopical examinations of the Fallopian tubes after their removal in these cases nearly always showed the presence of indigo or carmine particles, while in several cases the pigment was found on the peritoneal surfaces of the broad ligament, mesosalpinx, and the fimbriæ of the Fallopian tubes. This shows that the particles are carried from the os uteri below, right through the uterus and the Fallopian tubes, to the pelvic peritoneum. In the 22 cases thus investigated in which pigment was placed in contact with the os uteri previous to operation, there were only four in which the pigment was not subsequently found in the Fallopian tubes. And of these four cases, it is interesting to note that one was a case of double hydrosalpinx, in which any connection with the uterine cavity was difficult or obliterated; in one the indigo had been inserted only 16 hours before operation; and in the remaining two cases the indigo was inserted on the first day of the establishment of the menstrual flow, and probably failed to enter the uterus.

### The Upper Intestinal Tract.

In four cases of gall-bladder fistula following the removal of gall-stones, Mr. Bond administered to

* The Address in Surgery delivered at the seventy-third annual meeting of the British Medical Association by Charles John Bond, F.R.C.S., Eng.

the patients indigo by the mouth in cachets containing about 5 grs. The bile from the fistula was then examined for pigment, and in every case, after varying intervals, it was found.

The Urinary Tract.

In a case of fistula in the lumbar region following upon nephrectomy, the introduction of indigo into the bladder was followed by the appearance of the pigment in the discharge escaping from the fistula. Other experiments on patients who had at the time open supra-pubic cystotomy wounds, showed that particles of pigment introduced into the external urinary meatus were conveyed along the urethra and appeared in the urine coming from the wound.

Mammary Gland.

Not the least interesting of Mr. Bond's experiments were those by which he showed the transference of indigo particles from the surface of the nipple into the depths of the mammary gland. This process he demonstrated on several occasions after the removal of a breast for malignant disease. Although before accepting them as scientific facts it is necessary to await their verification at the hands of other investigators, it is not altogether unprofitable to meditate upon the benefits which are likely to accrue to medical and surgical practice if Mr. Bond's observations prove true. In the first place, if particles of indigo can traverse the large intestine or the ducts of the mammary gland in a direction contrary to the usual current in these canals it is probable that micro-organisms can do likewise. This may be the true explanation of the ascending infection which occurs in urinary stasis, in biliary stasis, and so forth. It may account for the frequent occurrence of salpingitis and pelvic peritonitis as a result of gonorrhœa in the female. And without mentioning other of the numerous "ascending infections" which are such a vexation to mankind, it is certain that, if Mr. Bond's explanation of their mechanism stands the test of time, much may be done in the future by means of surgical cleanliness to prevent this kind of microbic invasion.

